# Murdock’s Liquid Food

**Published:** 1888-07

**Authors:** 


MURDOCK'S LIQUID FOOD, ETC.—The proprietors of these food preparations have sent us beautiful samples of the liquid food, and it has attained a large reputation with both the public and the profession. For nourishment in low states of the system, it has proven very convenient and useful. Their raw food suppositories are highly recommended to nourish the patient in cases of vomiting from pregnancy and other conditions where the stomach will not appropriate the food.

DR. K. P. MOORE, of Macon. writes: "I have several quite interesting cases which I intend writing up soon. One, a case of vesico-vaginal fistula, very large, so much so as to allow almost complete eversion of bladder through rent. The patient conceived while in this condition. I operated at four and a half months of pregnancy with only partial success. A week or ten days after removing stitches she aborted. I operated the second time about two weeks ago, with entire success; patient now up. I promise you a report of this case soon. I will also give you an occasional report fiom the Macon Medical Society."

